# Comparing Two Commercially Available Diabetes Apps to Explore Challenges in User Engagement: Randomized Controlled Feasibility Study

**DOI:** 10.2196/25151

**Published:** 2021-06-16

**Authors:** Alita Maharaj, David Lim, Rinki Murphy, Anna Serlachius

**Affiliations:** 1 Department of Psychological Medicine Faculty of Medical and Health Sciences University of Auckland Auckland New Zealand; 2 Department of Medicine Faculty of Medical and Health Sciences University of Auckland Auckland New Zealand

**Keywords:** type 2 diabetes, mobile apps, diabetes, self-management, user engagement, app, mHealth, randomized controlled trial, intervention, efficacy

## Abstract

**Background:**

Diabetes apps represent a promising addition to face-to-face self-management interventions, which can be time and resource intensive. However, few randomized controlled trials have evaluated the efficacy of diabetes apps, in particular as a stand-alone intervention without additional clinical support.

**Objective:**

We used a feasibility randomized trial design to investigate differences in user engagement between 2 commercially available apps (free versions of Glucose Buddy and mySugr) over 2 weeks in adults with type 2 diabetes. Feasibility was assessed based on recruitment uptake, adherence to the diabetes apps, and follow-up rates. We also hypothesized that the diabetes app mySugr would demonstrate higher user engagement at follow-up due to its use of gamification. We also predicted higher user engagement would be associated with improved self-care behaviors and illness beliefs.

**Methods:**

Adults with type 2 diabetes attending outpatient diabetes clinics in Auckland were recruited and randomized (1:1 without blinding) to use either the Glucose Buddy or mySugr diabetes apps. User engagement, self-care behaviors, and illness beliefs were measured 2 weeks after baseline. Spearman rank correlations, Mann-Whitney tests, and Wilcoxon signed-rank tests were used to explore associations between the outcome measures and to investigate possible changes between and within groups. Six participants were interviewed to further explore acceptability and usability.

**Results:**

In total, 58 participants (29 per group) completed the 2-week follow-up, of whom only 38 reported using the apps (Glucose Buddy: n=20; mySugr: n=18). Both groups reported low engagement (Glucose Buddy: median 4 days; mySugr: median 6.5 days; *P*=.06; use for both groups: median 10 minutes). No changes were observed in self-care or illness beliefs in either group. Out of the self-care behaviors, only blood glucose testing was significantly associated with minutes of app use (*P*=.02). The interviews suggested that although both apps were deemed acceptable, they were generally viewed as time-consuming and too complicated to use.

**Conclusions:**

Low engagement with both Glucose Buddy and mySugr reflect the challenges associated with engaging users with diabetes apps. Due to low engagement and loss to follow-up, the changes in outcome measures should be interpreted with caution. The results highlight the need for more clinical support as well as involvement from end users and behavior change specialists in order to incorporate evidence-based behavior change techniques to motivate and provide value to users.

**Trial Registration:**

Australia New Zealand Clinical Trials Registry ACTRN12618000424202; https://www.anzctr.org.au/Trial/Registration/TrialReview.aspx?id=374671

## Introduction

The World Health Organization (WHO) estimates that diabetes affects over 400 million people [1]. Of all people with diabetes, an estimated 90% have type 2 diabetes (T2D) [2]. Treatment for T2D is multifaceted and includes modifying health behaviors, such as diet and physical activity, checking blood glucose levels, and adhering to medication. In New Zealand, diabetes affects approximately 241,000 people, with the majority having T2D [3]. T2D disproportionately affects people of Māori, Pasifika, and South Asian descent and is also more common among people residing in more socioeconomically disadvantaged regions in the country [4].

Several factors influence an individual’s adherence to a diabetes treatment regimen, including economic and sociocultural factors as well as beliefs and cognitions regarding their illness [5-7]. For example, several studies have found that illness perceptions (ie, the cognitive and emotional representations that people have of their illness) influence how people with T2D cope with their illness and the degree to which they adhere to their treatment regimen [8,9]. Unlike sociocultural factors, illness beliefs are modifiable [10] and may therefore represent a promising approach for improving self-care behaviors and glycemic control in diabetes [11].

Self-management education for T2D generally involves face-to-face interactions between individuals and health care professionals, who provide instructions and limited cognitive and behavioral strategies (within the resource limitations of the health care environment) to help people to manage their diabetes [12,13]. Mobile technologies, including commercially available diabetes apps, represent a more scalable and potentially more cost effective alternative to traditional interventions, offering a means of improving T2D management by expanding the reach of health care services and improving individuals’ access to health-related information and interventions [14,15].

Commercially available diabetes apps vary in the number and type of self-management behaviors they support [16]. The most commonly found features include logging of health information—including blood glucose levels, weight, physical activity, blood pressure, and dietary intake—educational modules, and insulin bolus calculators [16-18]. A large number of apps also provide some form of feedback to the user, most commonly as a graphical summary of their data or as a phone notification [17,18]. Some apps may also integrate directly with select blood glucose monitoring devices, allow data to be exported in various formats to be shared with third parties, or connect users directly with health care providers (HCPs) for feedback [16,18].

Reviews have increasingly suggested that diabetes apps may improve glycemic control and self-care behaviors in people with diabetes [17,18], possibly by facilitating the monitoring of self-care behaviors (eg, blood glucose monitoring) [19]. However, the findings of such reviews have several limitations that make it difficult to generalize to the wider T2D population. For example, detailed analyses of efficacy in the context of ethnicity, gender, and disparity in health literacy remain limited [20,21]. In addition, despite commercially available apps having arguably the largest user base, there is still a lack of studies that measure user engagement of commercially available diabetes apps [16,22-24]. “User engagement” comprises both frequency and duration of technology use, along with the users’ overall experience of the technology [25]. It is therefore not surprising that user engagement is thought to be integral to whether or not a digital intervention is effective [26]. Furthermore, there is a lack of theoretical input into the development of health apps aimed at changing health-related behavior. The vast majority of health-related apps are not theory-based, and their efficacy for improving health-related outcomes has not been sufficiently tested [27-31]. Finally, despite the large number of commercially available diabetes apps, there are few randomized controlled trials investigating the efficacy of these apps, especially studies which explore the efficacy of the app without additional clinical support [32-34].

A promising approach for increasing user engagement is gamification [35-37]. The concept of gamification is arguably context specific but is generally defined as the use of elements (eg, score systems, avatars, challenges, awards) commonly linked with video games in a nongaming setting [38,39]. It is suggested that when integrated into digital health interventions, these elements may increase motivation and learning [35,36,40] (see Landers et al [40] for an overview of the psychological theories behind gamification). However, evidence for the effectiveness of gamification in digital health interventions is mixed [36], particularly as it concerns whether gamification can improve health or psychological outcomes [35].

This study aimed to address these gaps in the literature by conducting a randomized controlled feasibility study to explore user engagement of 2, free, commercially available diabetes apps (Glucose Buddy and mySugr) that function without additional clinical support. We were specifically interested in whether the aspect of gamification (present in the mySugr app) could increase user engagement and thereby influence self-care behaviors. We also wanted to explore whether there was a relationship between user engagement and adherence to self-care behaviors. These apps were chosen, as they are both popular, have high user ratings on both iOS and Android [41,42], and contain functions deemed most useful by users of diabetes apps [43], with 1 app being explicitly based on gamification [44]. We hypothesized that mySugr, by virtue of its use of gamification, would be rated as more engaging than would Glucose Buddy and would demonstrate between-group improvements in self-care behaviors. We also hypothesized that higher user engagement would be associated with improved self-care behaviors at follow-up.

## Methods

### Participants

Participants were recruited from Auckland diabetes outpatient clinics between April 24, 2018, and July 24, 2018, and were randomized to trial 1 of 2 free apps (Glucose Buddy or mySugr), with follow-up after a 2-week trial period. Eighty-nine patients with T2D consented to participate and provided baseline data. This sample size was considered adequate to assess feasibility and conduct a preliminary evaluation of differences in user engagement between the 2 diabetes apps. Ethics approval for the study was obtained from the Health and Disability Ethics Committee on February 26, 2018 (reference #18/STH/43), and the study was prospectively registered with the Australia New Zealand Clinical Trials Registry on March 23, 2018 (ACTRN12618000424202). Inclusion criteria required that participants were 18 years or older; had a diagnosis of T2D; had the ability to speak, read, and write in English, and provide informed consent; and owned an iOS or Android smartphone capable of downloading apps.

### Procedure and Randomization

After completing baseline questionnaires, participants were randomly assigned 1:1 to parallel groups (Glucose Buddy or mySugr) using a computer-based random number generator. Blinding was not used. Randomization was done using sealed envelopes labeled with sequential study numbers. After randomization, AM helped the participants download the app onto their phone to use for 2 weeks. After the 2-week trial, participants were asked to complete a set of follow-up questionnaires online or were posted a hard copy of the questionnaires. Participants who completed the follow-up questionnaires received a NZ $20 (US $14.48) voucher to thank them for their time.

### Intervention Groups

#### Glucose Buddy

The diabetes app, Glucose Buddy, is a commercially available app developed by Azumio Inc. The free version of the app was used. The app facilitates the manual entry of information pertaining to various self-care behaviors and other health parameters, including exercise, diet, blood glucose, medications, blood pressure, and glycated hemoglobin (HbA_1c_). Users can track trends in these behaviors over time. The glucose tab allows users to log blood glucose levels, carbohydrates and food, and medication in 1 entry. Colour-coded graphs assist with monitoring blood sugar levels and medication. The app also has a large food database, and users can manually enter or scan the barcode of food items to record calorie and nutrition information. Participants were asked to use the app at their own pace, with no minimum or maximum requirements for usage time or features used.

#### mySugr

mySugr is a diabetes app developed by mySugr GmbH (acquired by Roche in 2017). The free version of the app was used. The mySugr app facilitates the manual input of information relating to self-care behaviors and other health parameters, including exercise, diet, medications, blood glucose, HbA_1c_, and blood pressure. Users can also track trends in these behaviors over time and set a target range for their blood glucose levels. A traffic light system facilitates monitoring of blood sugar levels, whereby entries falling within the target range are green and entries falling outside this range are red or orange depending on the values set by the user. A graph at the top of the home screen shows diet, exercise, medication, and blood glucose levels. Additionally, gamification is incorporated into all the key features of the app through the virtual avatar called the “diabetes monster.” Users can “tame” their diabetes monster based on their entries, which earns them points. Again, there were no minimum or maximum requirements for usage time or features used.

### Measures

Demographic details of age, sex, ethnicity, education, and employment status were collected at baseline through self-report. Other relevant information, including diabetes duration, HbA_1c_ levels at baseline (time of recruitment), and current diabetes treatments (including for comorbid conditions), was also obtained from patient medical records.

We used self-report questionnaires to examine user engagement, adherence to the diabetes apps, and changes in self-care behaviors and illness beliefs. To determine feasibility, we examined recruitment uptake, self-reported adherence to the diabetes apps, and follow-up rates. Due to the feasibility trial design, we did not specify primary or secondary outcomes.

#### User Engagement

User engagement was measured using an adapted form of the Mobile Application Rating Scale [45]. The original instrument was created for researchers, app developers, and health professionals to rate the quality of health apps. The current study used a simplified, user version of the Mobile Application Rating Scale (uMARS), which was designed for app users to complete [46]. The uMARS comprises 4 subscales: engagement, functionality, aesthetics, and information quality. In total, these subscales include 16 items. All items are rated on a 5-point Likert scale, where 1 indicates the app is unsatisfactory in that area and 5 indicates the app is excellent in that area. Mean scores are calculated for each subscale, and a total uMARS mean score is calculated by adding the mean scores for each of the subscales and dividing the total by 4. The uMARS demonstrates good internal reliability for both the whole instrument and for the individual subscales within the instrument [46]. The Cronbach α for the instrument in the present sample was .95.

Two additional questions were also included to measure users’ level of adherence with the apps. These were as follows: “In the last 14 days, on how many days did you use the app?” and “On the days that you used the app, approximately how many minutes did you spend using the app?”

#### Self-Care Behaviors

Self-care behaviors were assessed using a modified form of the Summary of Diabetes Self-Care Activities (SDSCA) [47]. This scale measures many facets of diabetes self-management: blood glucose testing, exercise, foot care, smoking, and general and specific diet and medication-taking behaviors. As diabetes self-management is multifaceted, this instrument allows scores for each component to be calculated individually. This study focused on self-care behaviors, and so to minimize participant burden, the 14 extra items from the expanded version of the SDSCA were omitted and only the first 7 subscales pertaining to self-care behaviors were included.

All 7 subscales were scored as the number of days per week participants engaged in a particular self-care behavior (eg, followed a healthy eating plan) on a scale of 0 to 7 days. Medication adherence was assessed with 1 item: “On how many of the last seven days did you take your recommended diabetes medication?”; the total number of days was then used to indicate participants’ medication adherence behavior. The general diet, exercise, blood glucose testing, and foot care subscales all contained 2 items each. Means for each of the subscales were calculated with higher numbers signifying better adherence to the behavior in the previous 7 days. The specific diet subscale was also made up of 2 items; however, the authors of the scale advised that these items be scored individually due to the low interitem correlations for the subscale [47]. Additionally, the specific diet item, “On how many of the last seven days did you eat high fat foods such as red meat or full-fat dairy products?” was reverse coded in scoring, as it indexed less healthy dietary behavior. Finally, smoking status was scored as a yes or no response to the question, “Have you smoked a cigarette—even one puff—during the past seven days?” This section further asked participants that responded yes to specify how many cigarettes they smoked on an average day. The SDSCA demonstrates adequate reliability and validity across T2D populations [48,49].

#### Illness Beliefs

Illness beliefs were measured using the Brief Illness Perceptions Questionnaire [50]. This scale has 9 items that assess cognitive and affective beliefs about illness. The cognitive items assess individuals’ beliefs relating to the timeline, identity, controllability, consequences, and causes of the illness. The remaining items assess individuals’ concern, understanding, and emotional representations of their condition. Furthermore, 8 of the 9 items are rated on a 0 to 10-Likert scale, where 0 represents the lowest score and 10 represents the highest score. The instrument shows satisfactory reliability and validity across a range of chronic conditions, including T2D [51].

### Statistical Analyses

**Figure 1:**
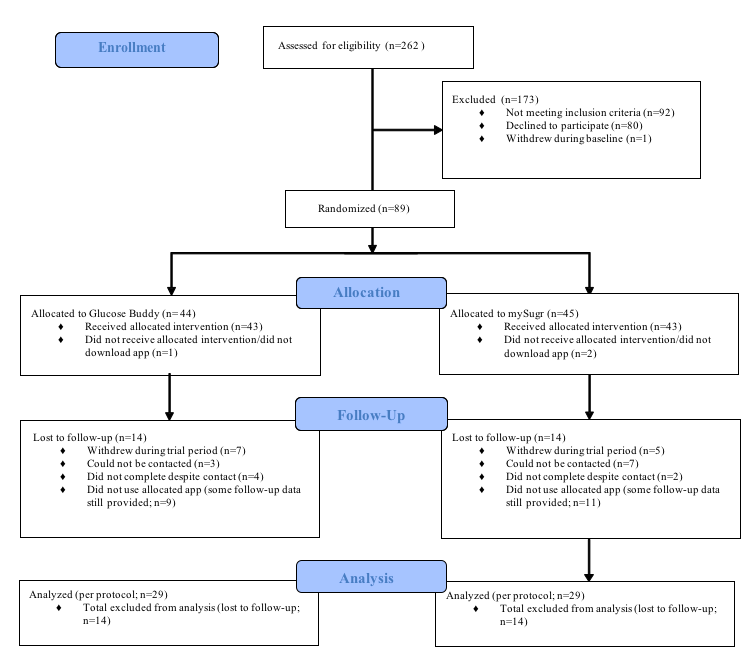
Consolidated Standards of Reporting Trials (CONSORT) diagram of participant involvement.

The study was designed to explore the feasibility, acceptability, and possible differences in user engagement, self-care behaviors, and illness beliefs between the 2 app groups. Preliminary analyses were conducted to examine whether the data complied with parametric assumptions. The key outcome variables were not normally distributed; therefore, Mann-Whitney tests were used to examine differences between the 2 groups in user engagement, self-care behaviors, and illness beliefs at follow-up. Wilcoxon signed-rank tests were also used to check for changes in participants’ self-care behaviors and illness beliefs from baseline to follow-up in each group. Spearman rank correlations were conducted to explore the relationships between user engagement and self-care behaviors at follow-up. Due to significant loss to follow-up (Figure 1), missing data were not included in the analyses and per protocol analyses were conducted.

The qualitative data obtained from the interviews were assessed using quantitative content analysis. Quantitative content analysis involves assessing how participants use language to describe their experiences and includes systematically allocating content into numerical categories [52]. In this study, quantitative content analysis was used to further explore participants’ experiences of using the apps, which included assessing the acceptability and usability of the apps and exploring views on how the apps could be improved.

## Results

Overall, 89 patients agreed to participate and provided baseline data. Of these, 31 were lost to follow-up and did not complete any of the follow-up questionnaires. Ultimately, 58 participants (29 per treatment arm) completed all assessments and were included in the final analyses (Figure 1).

### Baseline Characteristics

The sample at baseline (N=89) was mostly male (58/89, 65%), ranging in age from 28 years to 80 years with a mean age of 53 years (SD 11.99). The majority identified as Indian (30/89, 34%) or Pasifika (22/89, 25%), had done some tertiary level study (42/78, 54%), were married (60/88, 68%), and were employed (69/89, 78%; Table 1).

With regard to clinical characteristics, the mean age at which participants were diagnosed with T2D was 43 years (SD 11.28). On average, participants had been diagnosed with T2D for 9.9 years (SD 6.93) and had a mean BMI of 33.8 kg/m^2^. HbA_1c_ levels for participants at the time of recruitment ranged from 39 mmol/mol (5.7%) to 111 mmol/mol (12.3%), with a mean HbA_1c_ level of 68.4 mmol/mol (8.4%). Approximately half the sample consumed alcohol (47/88, 53%), while a considerably smaller percentage of participants smoked (9/88, 10%). Most participants had other long-term illnesses together with their T2D (70/89, 79%), with hypertension (63/89, 71 %) being the most commonly reported comorbid condition. Metformin was the medication participants were predominantly using to manage their T2D (80/88, 91%).

Equal numbers of participants owned an iPhone (38/87, 44%) or Samsung (38/87, 44%) smartphone, while the remaining participants (11/87, 13%) owned other Android smartphones, such as Sony, Huawei, or Oppo. Most participants (82/89, 92%) reported using apps on their phone and slightly more than one-third of the sample (33/89, 37%) reported using health apps.

**Table 1:** Baseline characteristics of participants (N=89).

Characteristic		Glucose buddy (n=44)	mySugr (n=45)	*P* value	
Age (years), mean (SD)		53.15 (11.06)	52.59 (12.96)	.83	
**Sex, n (%)**					.46
	Male	27 (61.4)	31 (68.9)		
	Female	17 (38.6)	14 (31.1)		
**Ethnicity, n (%)**					.90
	New Zealand European	6 (13.6)	9 (20)		
	Māori	4 (9.1)	5 (11.1)		
	Pasifika	12 (27.3)	10 (22.2)		
	Indian	16 (36.4)	14 (31.1)		
	Other	6 (13.6)	7 (15.6)		
**Relationship status^a^, n (%)**					.17
	Single	7 (15.9)	11 (25)		
	In a relationship	3 (6.8)	7 (15.9)		
	Married	34 (77.3)	26 (59.1)		
**Education^b^, n (%)**					.16
	Secondary education	14 (37.8)	22 (53.7)		
	Tertiary education	23 (62.2)	19 (46.3)		
**Employment status, n (%)**					.95
	Employed	34 (77.3)	35 (77.8)		
	Unemployed	10 (22.7)	10 (22.2)		

^a^Missing data for 1 participant (n=88).

^b^Missing data for 11 participants (n=78).

### Feasibility and Attrition to the Intervention

It took 2 months to recruit 89 participants: 31 participants were lost to follow-up after completing the baseline questionnaires, and 20 participants did not use their allocated app but completed the self-care behaviors and illness beliefs measures at follow-up and were included in the final analyses. There were significant differences between individuals who completed the study (n=58) and those lost to follow-up (n=31) for sex (*χ*^2^_1_=4.59; *P*=.03), with the proportion of men not using their allocated app (38/51, 75%) being greater than the proportion of women (13/51, 26%). There were also significant differences for ethnicity (*χ*^2^_1_=11.43; *P*=.02), with European New Zealanders more likely to complete the study compared to the other ethnic groups. Of those participants who dropped out, a greater proportion identified as Indian (24/51, 47%) compared with other ethnicities.

### User Engagement and Self-Care Behaviors

Out of the 58 participants who completed the study, only 20 participants in the Glucose Buddy group and 18 participants in the mySugr group reported using the apps during the trial (Table 2). Self-reported user engagement was low in both groups (Glucose Buddy: median 4 days; mySugr: median 6.5 days; *P*=.06; use for both groups: median 10 minutes). The median uMARS score was 3.37 for Glucose Buddy and 3.36 for mySugr (uMARS scale 1-5).

There was little evidence to suggest any between-group differences in self-reported user engagement scores between the 2 app groups or in self-reported adherence to the diabetes apps (Table 2). No improvements were found in self-care behaviors or illness beliefs from baseline to follow-up in either group (Table S1, Multimedia Appendix 1).

In regard to associations between user engagement and self-care behaviors, no significant relationships were found between number of days of app use and any of the self-care behaviors at follow-up (Table 3). Blood glucose testing was positively and moderately related to minutes of app use (Spearman ρ=0.37; *P*=.02). There were no significant relationships found between total uMARS scores and self-care behaviors (Table 3).

**Table 2:** Self-reported user engagement of the 2 apps.

Measure	Glucose Buddy, median (n=20)	mySugr, median (n=18)	*P* value
Days used	4.00	6.50	.06
Minutes used	10.00	10.00	.43
Engagement	3.20	3.30	.39
Functionality	3.25	3.63	.16
Aesthetics	3.33	3.50	.58
Information	3.50	3.75	.47
Total uMARS^a^ score	3.37	3.36	.89

^a^uMARS: user version of the Mobile Application Rating Scale.

**Table 3:** Spearman rank correlations between user engagement (days used, minutes used, total uMARS^a^ score) and self-care behaviors at follow-up.

Measures	Days used	Minutes used	Total uMARS score
General diet	–0.04	0.21	–0.04
Fruit and vegetable consumption	–0.14	0.27	0.09
High-fat foods consumption	–0.03	0.02	–0.18
Exercise	–0.15	–0.06	0.17
Blood glucose testing	–0.07	*0.37* ^b^	0.34
Foot care	0.004	0.04	0.07
Medication adherence	–0.08	0.16	0.16

^a^uMARS: user version of the Mobile Application Rating Scale.

^b^Italics indicate *P*<.05.

### Interviews

After the 2-week trial, 6 participants (3 from each app group) were interviewed over the phone after about their experience of using the diabetes app (Table 4). A diverse group of participants who reported having used the apps were selected for the interviews in order to obtain a variety of viewpoints. Of the interviewees, 3 were female and 3 were male and aged between 29 and 58 years; 1 participant was New Zealand European, 2 participants were of Pasifika descent, 2 were of Indian descent, and 1 was of Chinese descent. Regarding medication use, 1 participant’s regimen included both oral diabetes medication and insulin, and the remaining 5 participants reported taking oral medication only. Additionally, 4 of the 6 participants reported being advised to test their blood sugar regularly, and the remaining 2 participants reported not being required to test their blood sugar regularly as part of their T2D self-management. Feedback was grouped into positive experiences, negative experiences, most frequently used functions, and suggestions for improvement (see Table 5 for a summary of the participants’ feedback).

**Table 4:** Illustrative interviewee responses.

Feedback category by respondent			Illustrative quote			
**Positive experiences using the app**						
	Male, 30 years, mySugr app	“I was curious and excited during the demonstration…good initial impression.”				
	Female, 50 years, Glucose Buddy app	“Looked great when going through initially.”				
	Male, 49 years, mySugr app	“Yes, certainly I would download such an app…Could be subsidized? I am willing to pay if it’s worthwhile.”				
	Female, 50 years, mySugr app	“Keeping track of glucose testing, keeping track of medication.”				
	Female, 29 years, mySugr app	“Liked the diabetes monster.”				
**Negative experiences using the app**						
	Female, 58 years, Glucose Buddy app	“Diet and calories is good and what I found most useful and necessary, but I don’t know how to calculate, for example, how many calories in a piece of meat or a bowl of rice?”				
	Female, 29 years, mySugr app	“A bit confusing sometimes, don’t understand all of it. Carbs a bit confusing.”				
	Male, 49 years, mySugr app	“Converting carbs challenging. Food descriptions take a lot of time.”				
	Female, 50 years, Glucose Buddy app	“I wanted to use it and tried to use it a few times, but I kept getting stuck on the medication page. I often couldn’t navigate to other pages.”				
	Male, 30 years, mySugr app	“Occasionally froze, a bit slow.”				
	Female, 58 years, Glucose Buddy app	“So many features. Lots of things I need to know before I can use it. I have well-controlled diabetes, so only need simple monitoring functions.”				
	Male, 49 years, mySugr app	“I want to do it quite quickly, but this app has too many things…too much information and too many questions. Could be simplified.”				
	Male, 45 years, Glucose Buddy app	“Recording what I was already doing, so wasn’t super useful. Became too much of a chore. Not interesting enough. No motivation.”				
	Male, 49 years, mySugr app	“Current app is too much like a log book and not engaging enough.”				
**Most frequently used functions**						
	Male, 30 years, mySugr app	“Blood glucose result most useful. Logging everything...Keeping track of numbers.”				
	Female, 29 years, mySugr app	“I liked putting my blood glucose test in. Blood glucose test was the most useful feature.”				
	Female 58 years, Glucose Buddy app	“Advice on diet—sample advice. You should be eating this sized bowl of carbs, this amount of fruit and vegetables, this much butter and fats. Videos. Suggestions of age-appropriate activities and how to do it safely.”				
	Female, 29 years, mySugr app	“Tips on exercise and food and nutrition. Good meals, new workouts would help a lot. Things you can do at home if you don’t have time to go out.”				
**Suggestions for improvement**						
	Female, 58 years, Glucose Buddy app	“Measure food and activities more regularly. See pattern between things, like food and activity and how it affected my blood sugar.”				
	Male, 49 years, mySugr app	“Should be able to set goals, for example, identify how many carbs you can eat per day and note down how much you’ve consumed at breakfast and how much you could still consume throughout the rest of the day. Same for activity—record steps taken so far and how many more to take to meet goals. Goals per day or per week.”				
	Male, 45 years, Glucose Buddy app	“People that have had diabetes for two or three years, we know about eating and testing blood sugars, but you have so many things going on you forget, so it would be helpful to have regular reminders that are relevant.”				
	Male, 49 years, mySugr app	“Something user-friendly and quick to enter. Simplify the current app and make it easier to navigate.”				
	Male, 45 years, Glucose Buddy app	“Way that smartphone can talk directly to glucose meter. Glucose meter only remembers one month’s readings and then records over, so would like to have it send readings directly to smartphone. Communication between existing apps, for example this app and Google Fit.”				

**Table 5:** Summary of participants' feedback and suggestions for improvement (n=6).

Feedback category	mySugr	Glucose Buddy
Positive experiences	Graphs, colorful images, good interface, easy to read, glucose log, diabetes monster	Graphs, glucose log, medication log, food log
Negative experiences	Commercial emails, confusing, time-consuming, carb calculator hard to use	Hard to use, advertisements to upgrade to paid version, calorie calculator hard to use
Most frequently used functions	Glucose log	Glucose log, medication log
Suggestions for improvement	Provide fun and relevant reminders, allow the ability to set goals, simplify the app, provide dietary advice, make it quicker to enter information	Provide dietary advice, provide exercise advice, give relevant reminders, add more visual content, provide feedback based on blood sugar levels, use more videos

### Positive Experiences of Using the App

All participants reported positive initial impressions of their respective app and reported a willingness to download a T2D smartphone app in the future and to pay for the app if they deemed the app to be valuable to them.

All participants reported finding both apps visually appealing. Four participants reported that they found the ability to monitor and log their blood sugar levels and produce graphs useful, and two participants also reported finding the medication and food logs useful. One participant also reported that they enjoyed the gamification aspect of mySugr.

### Negative Experiences Using the App

All participants reported finding some aspect of the app confusing to use, with 3 of the 6 participants expressing that they found calculating calories and carbohydrates to be particularly challenging. Half of the participants also reported experiencing some technical obstacles like difficulty navigating the app. One of the main drawbacks reported by 3 participants was the large amount of information that was required to be entered into the app to use it. Participants found this to be time-consuming and complicated to use.

Some participants also felt that the apps did not engage or motivate them enough. Five of the six participants mentioned that they had not learnt any new information, for instance, about diabetes or how to improve their self-care behaviors, and two participants also mentioned that they forgot about the app sometimes.

### Most Frequently Used Functions

Four of the six participants reported that logging blood glucose was one of the most useful features of the apps. Two participants reported that they used the medication tracker.

### Suggestions for Improvement

Four of the six participants expressed a desire for more education and advice around nutrition and physical activity. With regard to diet, participants reported being interested in receiving advice on the types and quantities of the various food groups that they should be consuming. Similarly, for exercise, participants wanted suggestions of new exercises that they could do, along with advice and encouragement.

A desire for other features, in particular feedback and goal setting, were also mentioned. Three participants talked about wanting to receive feedback based on the information they entered, and two participants discussed the importance of being able to set goals and see how they are progressing towards achieving their goals.

Another feature that all 6 participants mentioned was tailored reminders and notifications. Participants stated that having reminders relating to their self-care behaviors, such as a reminder to check their blood glucose levels or take their medication, would be useful, as would be reminders relating to their goals.

Three of the six participants also reported wanting an app that allowed for easy monitoring of their self-care behaviors and that was not too time-consuming. One participant reported that having a diabetes app that communicated with their glucose meter or existing apps would be helpful.

## Discussion

To our knowledge, this is the first study to compare user engagement and associated changes in self-care behaviors in 2 popular, commercially available diabetes apps as stand-alone interventions without additional clinical support. The results suggested that over a period of 2 weeks, participants spent a limited amount of time using the apps, only using the apps for a median of 4 days for Glucose Buddy and 6.5 days for mySugr. There was little evidence to suggest that participants found one of the apps to be more engaging than the other despite mySugr’s use of gamification. There were also no improvements in self-care behaviors or illness beliefs from baseline to follow-up in either group. Indicators of feasibility (including adherence to the diabetes apps and follow-up rates) suggest that expecting participants to engage daily with a diabetes app without additional clinical support may be unrealistic.

Regarding the qualitative data, although the apps were considered to be acceptable to participants based on favorable initial impressions of the apps and a willingness to download diabetes apps in the future, they also reported facing various challenges in terms of usability. Two main reported shortcomings were the time-consuming nature and complexity of the apps. Participants also reported wanting apps to include more education and advice about diabetes self-care behaviors like diet and exercise. Participants also reported wanting tailored reminders or notifications relating to T2D self-care behaviors in general and to their specific diabetes-related goals.

The quantitative results from this study contradict with many other recent trials of diabetes apps, which have demonstrated efficacy in improving self-care behaviors or glycemic control in patients with type 1 diabetes (T1D) or T2D [18]. For example, Kirwan and colleagues [53] tested Glucose Buddy (coupled with weekly text messages from a diabetes nurse educator) in adults with T1D and found significant improvements in glycemic control from baseline to 9 months compared to standard care but no changes in self-care behaviors. In contrast to our study, their study included weekly clinical support from a certified diabetes educator over the duration of the intervention. Other differences included their intervention being significantly longer (6 months), the inclusion of a standard care control group, and testing of the app in adults with T1D. It is likely that all these differences played a role in improving glycemic control, particularly the additional clinical support, which has been argued to be the deciding factor for whether diabetes apps are effective in improving diabetes management outcomes. This makes it difficult to untangle whether intervention effects are due to the app or the increased clinical contact [54].

In contrast, the qualitative findings regarding the reported challenges and complexity of information in both apps are consistent with previous findings, in particular for older adults living with diabetes who are likely to benefit from a smaller range of functions [55]. Usability is a key factor influencing whether users engage with apps or not, and in the this study, all 6 participants who were interviewed reported finding some aspect of the app confusing, which included difficulties with calculating calories and carbohydrates and issues navigating the app. In a survey of more than 900 individuals who had downloaded a health app, just under half of these people reported discontinuing use of the app, with one of the main reasons being that they felt the app was not easy to use [56]. Other research also suggests that users fail to engage at all or stop engaging with technology once they consider it to be too hard to use [25]. These findings highlight the importance of having user input during the design and development of health apps [57], as something that seems intuitive to app developers or researchers may not feel straightforward to users, particularly if they are not confident or are new to using apps.

Research on user engagement and design indicates that several elements influence engagement with technology, like gamification, interactivity, feedback, challenge, and novelty [23,58], yet commercially available diabetes apps do not seem to fully leverage these features. The principal behavior change technique used in both free versions of the apps was self-monitoring, with the primary function being the recording of diabetes-related self-care behaviors. Thus, it seems that neither gamification nor self-monitoring alone may be sufficient to engage users, and greater inclusion of other evidence-based behavior change strategies (eg, goal setting) and fully exploiting the unique functions of smartphone technology (eg, the ability to provide personalized feedback through real-time reminders) are needed to successfully increase engagement, modify illness beliefs, and improve self-care behavior.

The glucose log feature was reported by all 6 participants to be one of the most useful—if not the most useful—feature of the apps. This is in line with other research that also found the glucose log to be the most frequently used diabetes app feature in a sample of patients with T2D who reported using apps [43]. Of the self-care behaviors, only blood glucose monitoring was significantly associated with minutes of app use and also demonstrated a trend towards a significant association with overall user engagement. This suggests that individuals who used the app for longer also tested their blood glucose more regularly. This may also indicate that more adherent patients are more likely to use diabetes technology in general [59], including diabetes apps. This is worth exploring in future studies to determine how we can improve engagement of diabetes technology for people who are currently struggling with diabetes self-management. In addition, despite the increased use of commercially available health apps by HCPs, there is relatively little evidence or guidance available for HCPs to evaluate their quality and efficacy [22,60]. Future studies should also incorporate interviews with HCPs to gather their feedback on the clinical usefulness of diabetes apps in diabetes self-management.

Several limitations of this study should be noted, including the short follow-up period, lack of blinding, and the high levels of attrition. We also tested the free versions of the Glucose Buddy and mySugr apps instead of the pro or paid versions, with the latter being more likely to incorporate more features that enhance user engagement, like real-time feedback and reminders. However, we deliberately chose the free versions of each app, as people living with T2D in New Zealand often come from lower socioeconomic status backgrounds. We also did not include regular clinical support to help patients use and engage with the apps, as the focal point of the study was to explore how patients use and engage with diabetes apps without additional support from HCPs. Another limitation was the reliance on self-reported user engagement. Ideally, user engagement should include a range of user engagement metrics, including app analytics, which was not possible in this study. Furthermore, the qualitative data collected from 6 participants may not be representative of the study cohort and cannot be generalized to the wider T2D population. Another limitation is the lack of intention-to-treat analyses, which we were unable to conduct due to the missing data.

The strengths of this study include the randomized controlled design, the testing of 2 popular apps that are commercially available and free to use, and the recruiting of a diverse sample of people living with T2D. Future research comprising larger samples and higher rates of user engagement and interaction with apps would offer greater power for detecting possible between-group differences. Longer follow-up would also be beneficial in ascertaining whether diabetes apps could successfully encourage long-term behavior change. Finally, studies examining whether clinical support from HCPs leads to better outcomes compared with unsupported use of diabetes apps are needed. It remains to be seen whether larger trials testing diabetes apps without additional clinical support can sufficiently engage patients.

In conclusion, there was little evidence of between-group differences in user engagement, and neither app group showed improvements in self-care behaviors or illness beliefs after a median of 6.5 days and 4 days of use over 2 weeks for mySugr and Glucose Buddy, respectively. However, our findings suggest that individuals who used the apps for longer periods per day also tested their blood glucose more frequently. Overall, the results of this feasibility trial demonstrate how difficult it is for individuals with long-term conditions to engage with diabetes apps without additional clinical support. It also highlights the importance of having both patients’ and HCPs’ input during the app development process to ensure the app meets patients’ needs, both in terms of being user-friendly and engaging as well as targeting all self-care behaviors with appropriate behavior change techniques to support behavior change.

## Appendix

Multimedia Appendix 1 Median scores of self-care behaviours and illness beliefs by app group at follow-up.

Multimedia Appendix 2 CONSORT-eHEALTH checklist (V 1.6.1).

## Abbreviations

HbA_1c_: glycated hemoglobin
HCP: health care provider
SDSCA: Summary of Diabetes Self-Care Activities
T1D: type 1 diabetes
T2D: type 2 diabetes
uMARS: user version of the Mobile Application Rating Scale
WHO: World Health Organization
