# Supplementary material for: Comparing Two Commercially Available Diabetes Apps to Explore Challenges in User Engagement: Randomized Controlled Feasibility Study
Source: JMIR Form Res. 2021 Jun 16;5(6):e25151. doi: 10.2196/25151 (PMC8277312; doi:10.2196/25151)
Supplement: Multimedia Appendix 1 [file formative_v5i6e25151_app1.docx]

*Supplementary Table 1: Median scores, U, z and p-values of self-care behaviours and illness beliefs, by app group, at follow-up*

|  | **Glucose Buddy (n=20)** | **mySugr (n=18)** |  |  |  |
| --- | --- | --- | --- | --- | --- |
| **Variable** | ***Median*** | | ***U*** | ***Z*** | ***P*** |
| General diet | 4.75 | 5.00 | 169.50 | -.31 | .755 |
| Fruit and vegetable consumption | 5.00 | 5.00 | 177.50 | -.07 | .941 |
| High fat foods consumption | 5.00 | 4.00 | 108.00 | -2.15 | .052 |
| Exercise | 3.50 | 3.00 | 170.00 | -.29 | .769 |
| Blood glucose testing | 4.25 | 3.25 | 163.50 | -.49 | .626 |
| Foot care | 1.75 | 2.50 | 162.50 | -.52 | .606 |
| Medication adherence | 7.00 | 7.00 | 148.50 | -1.57 | .116 |
|  |  |  |  |  |  |
| Consequence beliefs | 5.00 | 6.00 | 178.50 | -.04 | .965 |
| Timeline beliefs | 8.00 | 8.00 | 163.50 | -.50 | .620 |
| Personal control beliefs | 6.50 | 5.85 | 172.00 | -.24 | .811 |
| Treatment control beliefs | 9.00 | 9.00 | 171.00 | -.28 | .782 |
| Identity beliefs | 4.50 | 3.00 | 150.00 | -.89 | .376 |
| Diabetes-related concerns | 8.50 | 7.50 | 159.50 | -.62 | .538 |
| Diabetes-related understanding | 8.50 | 7.50 | 158.00 | -.65 | .513 |
| Emotional effects | 5.00 | 5.00 | 145.50 | -1.02 | .309 |

*Note.* Only includes the participants who used the app at least once during the two-week trial (n = 38).
